# Feelings of Entrapment and Defeat Mediate the Association Between Self-Esteem and Depression Among Transgender Women Sex Workers in China

**DOI:** 10.3389/fpsyg.2019.02241

**Published:** 2019-10-04

**Authors:** Ruijie Chang, Huwen Wang, Rui She, Shuxian Zhang, Lhakpa Tsamlag, Qiuming Shen, Yue Shi, Zixin Wang, Joseph T. F. Lau, Ying Wang, Yong Cai

**Affiliations:** ^1^School of Public Health, Shanghai Jiao Tong University School of Medicine, Shanghai, China; ^2^Center for Health Behaviors Research, JC School of Public Health and Primary Care, The Chinese University of Hong Kong, Hong Kong, Hong Kong

**Keywords:** transgender women sex workers, depression, self-esteem, entrapment, defeat, mediating effect

## Abstract

**Background:**

Transgender women sex workers have a high prevalence of depression. Low self-esteem and subsequent involuntary subordination (characterized by feelings of defeat and entrapment) are well-documented risk factors for depression. The object of the present study was to investigate the mediating effect of feelings of entrapment and defeat on the relationship between self-esteem and depression among transgender women sex workers in China.

**Methods:**

A cross-sectional study was conducted in Shenyang and Guangzhou in 2017. Participants were 198 transgender women sex workers who completed a structured questionnaire assessing background characteristics, self-esteem, feelings of entrapment and defeat, and depression. Linear regression was used to test the mediation hypotheses.

**Results:**

Of participants, 25.25% exhibited high levels of depression. Self-esteem scores were negatively correlated with depression scores (*r* = −0.54, *p* < 0.05, *R*^2^_adj_ = 0.23), defeat scores (*r* = −1.68, *p* < 0.05, *R*^2^_adj_ = 0.31), and entrapment scores (*r* = −1.67, *p* < 0.05, *R*^2^_adj_ = 0.25). In the mediation hypothesis model, entrapment (*r* = 0.21, *p* < 0.05) and defeat (*r* = 0.08, *p* < 0.05) had a complete mediating effect on the relationship between self-esteem (Spearman’s *r* = −0.06, *p* = 0.36) and depression (*R*^2^_adj_ = 0.61).

**Conclusion:**

Feelings of entrapment and defeat mediated the association between self-esteem and depression. More focus is needed on monitoring feelings of defeat and entrapment among transgender women sex workers to mitigate the risk of depression.

## Introduction

Transgender women are individuals whose legal gender identity at birth is male, but who currently identify as female. Some transgender women live as females, wearing feminine clothing, using hormone treatment, and undergoing sex reassignment surgery (Cai et al., 2016). According to previous meta-analyses, about 24%–75% of transgender women in the United States are involved in sex work, while between 54% and 80% of transgender women in Asia are involved in sex work (Cohen et al., 2011; Poteat et al., 2015). Thus, transgender women sex workers are an important sector of the population of transgender women.

Depression is a serious mental disorder that not only impairs patients’ health and quality of life (Ferrari et al., 2013), but also places a substantial burden on families and societies (Jean-Pierre and Mike, 2011). Although few studies have examined depression among transgender women sex workers, estimates of depression in transgender women are as high as 62% (Hoffman, 2014), substantially higher than the estimated rate of depression in general population (2.06%) (Phillips et al., 2009).

The concept of self-esteem is defined as a person’s attitude toward themselves, and reflects how an individual evaluates their own self-concept (Aydm and San, 2011). According to Rosenberg (Rosenberg, 1965; Westaway et al., 2015), high self-esteem is a comprehensive positive evaluation of aspects of the self, including intellectual abilities, physical appearance, and social competence. They respect themselves and consider themselves worthy. If a person feels useless, unworthy and disconnected from other people, they may experience a lack of meaning in life. It is well established that low self-esteem and depression are related (Schmitz et al., 2003; Uher and Mcguffin, 2010). The vulnerability model proposes that low self-esteem leads to depression (Beck and Steer, 1990; Roberts and Monroe, 1992; Butler et al., 1994), while the scar model proposes that depression status causes one’s self-esteem to decrease (Coyne et al., 1998; Coyne and Whiffen, 1995). A 2013 meta-analysis of longitudinal studies covering 77 studies of depression and 18 studies of anxiety reported that the effect of self-esteem on depression was significantly stronger than the effect of depression on self-esteem (Julia Friederike and Ulrich, 2013).

One study tested various mediational models (Sturman, 2011) to clarify the nature of the relationship between self-esteem and depression. The findings identified personality, involuntary subordination, and mood as important factors, and provided new insights and methods regarding the mechanisms underlying depression. Studies of animal species with dominance hierarchies suggest that, after threats and non-lethal fighting, animals defeated in competition could be described as “depressed.” Gilbert and Allan (1998) used exploratory and confirmatory factor analysis to generate a model based on social rank theory and comprising social comparison, submissive behavior, feelings of defeat and entrapment, and involuntary subordination. Depression is a natural consequence of prolonged involuntary subordination (Brown et al., 1995; Gilbert and Allan, 1998; Gilbert et al., 2002; Rasmussen et al., 2011; Sturman, 2011). Previous studies identified that defeat and entrapment are closely associated with various types of human psychopathology, such as depression, anxiety, and suicide (Peter James et al., 2011; Alys Wyn et al., 2014).

Many studies have focused on the mechanism underlying the occurrence of depression with the aim of reducing the incidence of depression (Beck, 1987; Beck and Alford, 2009; Uher and Mcguffin, 2010). The relationship among self-esteem, entrapment, defeat, and depression is not clear yet. Since suicide and depression are both severe mental disorders, we searched for the literatures focused on the relationships among self-esteem, entrapment, defeat, and suicide. One study (Gooding et al., 2015) investigated the moderating effect of self-esteem on the relationships among feelings of defeat, entrapment, and suicidality in prisoners. Gooding et al. (2015) supposed that low levels of self-esteem heighten feelings of defeat and entrapment could inform efforts to reduce suicide rates. Although the result was contrary to predictions, it gives us some ideas. There is a study declared that defeat and entrapment appear to be promising variables for the study of depression (Gilbert and Allan, 1998). Is it possible that some mediating effects exist? In the current study, we sought to determine whether a similar causal path exists between self-esteem and depression, and whether entrapment and defeat mediate the relationship between self-esteem and depression.

There has been little research on transgender women sex workers, or on the relationships among self-esteem, depression, and feelings of entrapment and defeat in populations of transgender women sex workers. We hypothesized that there would be a mediational relationship among self-esteem, feelings of defeat and entrapment, and depression among Chinese transgender women sex workers. Evidence for such a relationship would have implications for mental health care for transgender women, and for future research in this area.

## Materials and Methods

### Participants and Eligibility Criteria

A cross-sectional study was conducted in Shenyang and Guangzhou, China, from April 2017 to July 2017. All participants were (1) at least 18 years old, (2) lived, worked and socialized in Shenyang or Guangzhou, (3) had the ability to understand the questionnaire, (4) had the ability to provide informed consent, and (5) self-identified as transgender women sex workers. An additional inclusion criterion was that participants had to have had intercourse with ≥1 male client in the last 3 months.

### Recruitment and Procedure

Snowball sampling was used to engage stakeholders from hard-to-reach vulnerable communities in this community-partnered and patient-centered outcomes research study (Magnani et al., 2005; Valerio et al., 2016). First, with the help of a non-governmental organization (NGO) dedicated to improving the physiological and psychological health of transgender women sex workers, we recruited 5–10 volunteers in each city as the “seeds.” Second, to locate harder-to-reach transgender women sex workers, we asked the “seeds” to identify other suitable people for participation in the study, thus aggregating the snowball until saturation was reached.

Participants were informed of the following points: that all data collected were anonymous and strictly confidential and would be used for research; that all investigations would be conducted in accordance with their own wishes and would not cause any adverse impact on them; respondents’ decision to participate or not would not affect the services they received at the institution. They could receive human immunodeficiency virus (HIV) rapid testing anonymously and freely if they wished. Participants were free to withdraw their participation at any time. Written informed consent was obtained before interviewing and HIV testing. A final total of 198 transgender women sex workers participated in this study. All participants completed the questionnaire during an anonymous face-to-face interview in a private room, and the assistant waited until participants answered all questionnaires and offered help if the meaning of questions was not clear. To measure potential demographic and psychosocial correlates of depression, we also included the following measures: (1) background characteristics, such as age, education level, marriage status, monthly income, duration of stay in Shenyang/Guangzhou, sexual orientation, ever received AIDS related education or not, HIV test results, willingness to accept voluntary counseling and testing (VCT), and smoking status in the past 30 days; (2) self-esteem status; (3) depression status; (4) defeat; and (5) entrapment. As part of the survey, we provided a finger-prick HIV rapid test (Alere Determine TM HIV-1/2 rapid HIV screening test, Alere Inc., Waltham (MA), United States; sensitivity = 99.75%; specificity = 100%) to all participants if they wished. Finger-prick HIV rapid test strips were provided by the local Center for Disease Control and Prevention. Among the participants, 18 transgender women sex workers did not undergo the HIV test although they did not know their current HIV status according to their self-reported results. We then determined that the percentages of participants who were HIV-positive, HIV-negative, and HIV-unknown were 27.8% (55/198), 63.1% (125/198), and 9.1% (18/198), respectively.

### Ethical Considerations

The study protocol and informed consent received ethical approval from the Ethics Committee of the School of Public Health of Shanghai Jiao Tong University, China. The study was carried out in accordance with the latest version of the Declaration of Helsinki. Trained survey researchers provided participants with consent forms describing the study objectives, risks, and benefits. Participants were able to withdraw from the study at any time and were allowed to use a pseudonym to sign the front page of the informed consent form. A face-to-face questionnaire and interview were conducted in a private room with the help of trained survey researchers. Each participant received monetary compensation (200 RMB, approximately 30 USD) after completing the questionnaire regardless of whether they underwent HIV-testing. Not participating in the survey did not affect the services received at the institution.

### Measures

#### Background Characteristics

The following background information was obtained: (1) age, (2) education level, (3) marital status, (4) monthly personal income, (5) length of residence in the city (Shenyang, Guangzhou), (6) sexual orientation, (7) received AIDS prevention education in the past 6 months or not, (8) willingness to accept HIV VCT, (9) any previous HIV VCT, (10) HIV status, and (11) smoking status in the past 30 days (Table 1).

**TABLE 1:** Background characteristics of transgender sex workers (*N* = 198).

**Sociodemographics**	**Number of participants**	**PHQ-9 scale score**			**Depression**	
	***n*(row%)**	**M(IQR)**	***t*/χ^2^**	***P***	***n*(row%)**	**ORu(95%CI)**
**Age**			0.34	0.84		
18∼27	57 (28.78)	5 (7.5)			16 (28.07)	1
28∼38	95 (47.98)	6 (6)			23 (24.21)	0.82(0.39−1.72)
39∼62	46 (23.24)	4 (9.25)			11 (23.91)	0.81(0.33−1.96)
**Education level**			3.39	0.34		
Primary or below	15 (7.58)	7 (7)			5 (33.33)	1
Junior secondary	83 (41.92)	5 (8)			24 (28.92)	0.81(0.25−2.63)
Senior secondary	50 (25.25)	5 (7)			8 (16.00)	0.38(0.10−1.42)
College or above	50 (25.25)	5 (8)			13 (26.00)	0.70(0.20−2.44)
**Marriage status**			6.55	0.04^∗^		
Unmarried	153 (77.27)	5 (7)			35 (22.88)	1
Married	11 (5.56)	2 (8)			1 (9.09)	0.34(0.04−2.73)
Divorced or widowed	34 (17.17)	7.5 (9.5)			14 (41.18)	2.36(1.08−5.15)^*^
**Monthly income**			6.6	0.04^∗^		
<450	46 (23.23)	6.5 (10.25)			18 (39.13)	1
450–900	96 (48.48)	5 (7)			22 (22.92)	0.46(0.22−0.99)
>$900	56 (28.28)	5 (7)			10 (17.86)	0.34(0.14−0.84)^*^
**Duration of stay in this city**			0.86	0.65		
Local	70 (35.35)	6 (7)			18 (25.71)	1
Migrant < 5 years	69 (34.85)	4 (6.5)			15 (21.74)	0.82(0.37,1.76)
Migrant > 5 years but not local	59 (29.80)	6 (10)			17 (28.81)	1.70(0.54−2.55)
**Sex orientation**			1.89	0.6		
Heterosexual	20 (10.10)	5.5 (6)			3 (15.00)	1
Homosexual	138 (69.70)	5 (8)			35 (25.36)	1.93(0.53−6.97)
Bisexual	32 (16.16)	5.5 (9.5)			9 (28.13)	2.22(0.52−9.45)
Others	8 (4.04)	5 (7)			3 (37.50)	3.40(0.52−22.41)
**Received AIDS related education**			0.40	0.53		
No	68 (34.34)	4.5 (8)			19 (27.94)	1
Yes	130 (65.66)	5.5 (6)			31 (23.85)	0.81(0.42−1.57)
**HIV testing result**			7.36	0.03^∗^		
Positive	55 (27.78)	6 (8)			21 (38.19)	1
Negative	125 (63.13)	5 (7)			24 (19.20)	0.39(0.19−0.78)^*^
Unknow	18 (9.09)	3.5 (10.25)	0.39	0.53	5 (27.78)	0.62(0.19−2.00)
**Willingness to accept VCT**						
No	16 (8.08)	3 (5.75)			3 (18.75)	1
Yes	182 (91.92)	6 (8)			47 (25.82)	1.51(0.41−5.53)
**Smoking status in the past 30 days**			0.16	0.98		
Never	79 (39.9)	6 (7)			20 (25.32)	1
Sometimes	35 (17.68)	7 (6)			8 (22.86)	0.87(0.34−2.23)
Often	26 (13.13)	4 (11)			7 (26.92)	1.09(0.40−2.97)
Everyday	58 (29.29)	5.5 (8)			15 (25.86)	1.03(0.47−2.24)
**Depression (PHQ-9)**						
Depressed (≥10 score)	50 (25.25)	–			–	–
Not depressed (>10 score)	148 (74.75)	–			–	–

*M, mean; IQR, interquartile range; VCT, voluntary counseling and testing; PHQ-9, Patient Health Questionnaire. ^∗^*p* < 0.05.*

#### Self-Esteem Scale Measure

The Rosenberg Self-Esteem Scale measures self-esteem status and comprises 10 Likert-type scale items (Mckay et al., 2014). The scale measures both positive and negative reflections of self-esteem. Each question has four response options ranging from 1 (strongly disagree) to 4 (strongly agree). The positive item response scores are 0 (never), 1 (seldom), 2 (sometimes), 3 (often), and 4 (always) and the negative item response scores are 4 (never), 3 (seldom), 2 (sometimes), 1 (often), and 0 (always). The lowest possible score is 10 and the highest possible score is 40 (Cronbach’s α = 0.81; range 12–40).

#### Depression Scale Measure

Although there are many valid tools to evaluate depression status, one systematic review concluded that the Patient Health Questionnaire (PHQ-9) is equal or superior to other depression instruments (Williams et al., 2002; Manea et al., 2015). The PHQ-9 is a nine-item, one-dimensional depression measure. Each question has four response options ranging from 0 (absolutely not) to 3 (almost every day). The possible total score ranges from 0 to 27; higher scores indicate more severe depression (Cronbach’s α = 0.90; range 0–27). Respondents who score ≥10 are diagnosed as exhibiting depression (Kroenke et al., 2001; Simon et al., 2007; Muñoz-Navarro et al., 2017).

### Defeat Scale

Gilbert and Allen’s Defeat Scale is designed to “capture a sense of failed struggle and losing rank” and evaluates the feelings of defeat over the previous 7 days (Gilbert and Allan, 1998). Response options are never, seldom, sometimes, often, and always. The 16-item scale contains two dimensions; total possible scores range from 0 to 64. Higher scores reflect more easily feeling defeat in daily life. Positive questions are scored positively and negative questions are scored negatively (Cronbach’s α = 0.90; range 0–61).

### Entrapment Scale

The one-dimensional Entrapment Scale comprises 16 items with five response options: 0 (not at all), 1 (light), 2 (medium), 3 (heavy), and 4 (serious) (Gilbert and Allan, 1998; Gilbert et al., 2002). Total possible scores range from 0 to 64. Higher scores indicate stronger feelings of entrapment (Cronbach’s α = 0.96; range 0–63).

### Statistical Analysis

Descriptive analysis and chi-square tests were used to describe sociodemographic characteristics and to compare the distribution of depressive symptoms, respectively. Participants were categorized into two groups based on PHQ-9 score: depressed (PHQ-9 score ≥ 10 score) and not depressed (PHQ-9 score < 10 score). The chi-square results revealed which background characteristics of transgender people increased the risk of depression. Univariate analysis was then performed using binary logistic regression to detect the association between background characteristics and depression. Subsequently, pairwise correlation analysis of the four scale scores was conducted to examine the relationship among the variables.

The mediation hypothesis was then examined using Baron and Kenny’s method (Baron and Kenny, 1986). The significance of the indirect effects was examined using the bootstrap procedure based on 5,000 samples to derive a bias-corrected 95% confidence interval (CI). Mean centering of the data for the four questionnaires was carried out; that is, the average score of each scale was set at 0. If the 95% CI of the indirect effect (path a^∗^b) did not contain 0, this indicated that the mediating effect was significant. As defeat and entrapment were interaction terms, we needed to consider whether the interaction between defeat and entrapment was a significant factor in this mediation hypothesis model. If the interaction between defeat and entrapment was not statistically significant, we excluded it from the mediation model. We used SPSS Statistics (version 23.0 for Windows, IBM, Armonk, NY, United States); *p*-values < 0.05 were considered statistically significant. In addition, all models were controlled for significant covariates in the chi-square test and univariate analysis.

## Results

### Background Characteristics

Participants’ age was categorized into three groups, 18–27, 28–38, 39–62 years, by range interquartile. A total of 25.25% of participants had attended college or above, 77.27% were not married, and 48.48% had a monthly income of 450–900 USD. The percentages of participants who were local, resident for <5 years, and resident for >5 years but not local were 35.35, 34.85, and 29.80%, respectively. A total of 69.70% reported homosexual orientation. Many participants had received AIDS-related education (65.66%). Only 9.09% reported an unknown HIV test result and 27.78% had been diagnosed as HIV-positive. Most participants (91.92%) were willing to accept VCT. A total of 29.29% had smoked daily in the past 30 days (Table 1). Of these background characteristics, depression was associated with marital status (*p* = 0.04), monthly income (*p* = 0.04), and HIV test result (*p* = 0.03). Participants with negative HIV test results were less likely to have depression than participants with positive HIV test results (ORu = 0.39, 95% CI = 0.19–0.78).

### Questionnaire Scores and Pairwise Correlation Analysis

As shown in Table 2, self-esteem was negatively correlated with depression, defeat, and entrapment. Depression was positively correlated with defeat and entrapment, and defeat was positively correlated with entrapment.

**TABLE 2:** Questionnaire scores and pairwise correlation analysis (*N* = 198).

	***n***		***t***		
	**Self-esteem**	**Depression**	**Defeat**	**Entrapment**
Self-esteem scale score	1			
Depression scale score	−0.434^∗∗∗^	1		
Defeat scale score	−0.553^∗∗∗^	0.687^∗∗∗^	1	
Entrapment scale score	−0.495^∗∗∗^	0.767^∗∗∗^	0.788^∗∗∗^	1
Mean	29.727	6.551	18.546	15.439
Standard deviation	4.519	5.699	13.954	15.578

*^∗∗∗^*p* < 0.001.*

### Testing the Mediation Hypothesis

**FIGURE 1:**
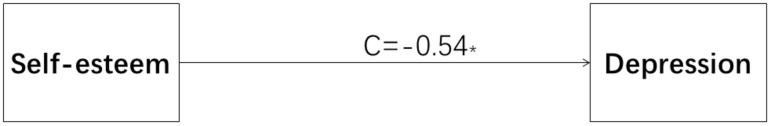
The relationship between self-esteem and depression among transgender women sex workers. ^∗^*p* < 0.05.

**FIGURE 2:**
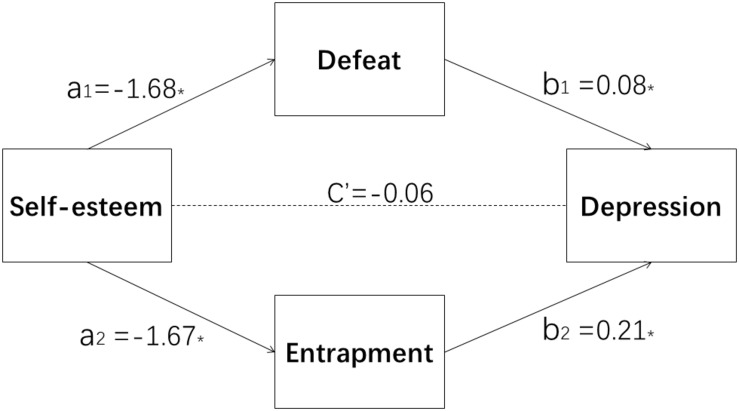
Feelings of entrapment and defeat mediate the association between self-esteem and depression among transgender women sex workers. ^∗^*p* < 0.05.

After adjusting for marital status, monthly income, and HIV test result, self-esteem was significantly correlated with depression (*B* = −0.54, *p* < 0.05, *R*^2^_adj_ = 0.23) (Figure 1). In the first mediation hypothesis model, the mediators included defeat, entrapment, and the interaction between defeat and entrapment. The interaction between defeat and entrapment had no effect on depression (*r* = 0.002, *p* = 0.18, *R*^2^_adj_ = 0.61), so the interaction between defeat and entrapment was excluded. In the second mediation hypothesis model, we conducted further analysis to determine whether the indirect effect of self-esteem on depression via entrapment and defeat was significant. Entrapment (*r* = 0.21, *p* < 0.05) and defeat (*r* = 0.08, *p* < 0.05) had a complete mediating effect on the relationship between self-esteem (*r* = −0.06, *p* = 0.36) and depression (Figure 2), *R*^2^_adj_ = 0.61. The total effect was −0.55, the indirect effect was −0.49, and the direct effect was −0.06 (Table 3).

**TABLE 3:** Results of mediation analysis (*N* = 198).

**Variable**	**Path c**		**Path c’ & b_1_ & b_2_**		**Path a_1_**		**Path a_2_**		**Path a_1_^∗^b_1_ + a_2_^∗^b_2_**
	**B**	**SE**	**B**	**SE**	**B**	**SE**	**B**	**SE**	**B**
Self-esteem	−0.54**^∗^**	0.08	−0.06	0.07	–	–	–	–	−0.49
Defeat	–	–	0.08^∗^	0.03	−1.68^∗^	0.19	–	–	
Entrapment	–	–	0.21^∗^	0.03	–	–	−1.67^∗^	−0.22	
*R*^2^_adj_	0.23		0.61		0.31		0.25		
*F*	9.23		34.857		13.62		10.44		

*^∗^After adjusting for marital status, monthly income, and HIV test result, *p* < 0.05.*

## Discussion

We conducted a quantitative study to elucidate the relationships between depression and socio-demographic characteristics, HIV risk, and mental health status among transgender women sex workers in Shenyang and Guangzhou. We found that many transgender women sex workers are likely to experience serious mental disorders, such as depression. Over a quarter of the participants (25.25%) suffered from depression, which is a substantially higher rate than that reported in the general Chinese adult population (2.06%) (Phillips et al., 2009). Thus, the results of our study indicated that transgender women sex workers commonly experience depression, suggesting that more effort should focus on resolving this issue.

In the current study, a survey of transgender women sex workers in Shenyang and Guangzhou, we found that marital status, monthly income (in RMB), and HIV test results were related to depression. Divorced or widowed transgender women sex workers were at higher risk of depression than unmarried transgender women (OR = 2.36, 95% CI = 1.08–5.15). The result showed consistence with previous studies (Noorbala et al., 2004, 2017; Mirzaei et al., 2019) which indicated that divorced and widowed women had higher frequency of depression and anxiety than single people. The impact of cultural and societal views and stressors due to separation and divorced and financial problems may contribute to mental disorders in divorced and widowed participants.

We found that participants who earned more than $900 USD per month had a significantly lower risk of depression (OR = 0.34, 95% CI = 0.14–0.84). Higher economic status may have protected people from depression because having a sufficient amount of money may have enabled them to have more control over their lives. This result in the current study was similar to findings reported in previous studies. One previous study found a relationship between income and depression among men who have sex with men in south Florida, as lower income may have disadvantaged individuals and make them more vulnerable to participation in risky sexual behavior (De Santis et al., 2008). Seelman et al. (2016) found that lower income was strongly associated with poorer general health and with multiple indicators of poor physical and mental health, including depression, anxiety, and suicidal ideation. Our result also indicated that HIV seronegative participants were less prone to depression than HIV seropositive participants (OR = 0.39, 95% CI = 0.19–0.78). Our results are consistent with these past study findings. Many people assume that a diagnosis of HIV/AIDS is almost equivalent to a death sentence, causing people living with HIV/AIDS to be more susceptible to depression (Rabkin, 2008). Eighteen participants did not receive HIV testing, although they did not know their HIV status according to their self-reported results. To respect participants’ privacy, we did not force them to accept HIV testing or ask them to give reasons for refusing. These participants may have already known about their HIV status or thought it unlikely that they were infected. This finding suggests that NGO workers should offer more health education and assistance regarding HIV testing.

After adjusting for these three variables, marital status, monthly income (in RMB), and HIV test results, feelings of entrapment and defeat completely mediated the relationship between self-esteem and depression. These findings suggest that addressing feelings of entrapment and defeat may help to protect this population from depression. Self-esteem reflects how people feel about themselves (Baumeister, 2000). Leary and Baumeister describe self-esteem as a “sociometer” that reflects an individual’s cognitive processing of self-information and positive cognitive evaluation. Self-esteem reflects how people feel about themselves (Baumeister, 2000). According to Rosenberg (1989), people with high self-esteem tend to admit that they are good enough but do not necessarily consider themselves superior to others. Overall, self-esteem influences people’s choices and attitudes toward their life (Julia Friederike and Ulrich, 2013). Some research suggests that social anxiety disorder is related to low self-esteem (Iancu et al., 2015). As individuals with low self-esteem tend to be sensitive to other’s judgment and criticism, they may prefer to hide their inner feelings and avoid contributing substantially to group tasks, choosing to protect themselves rather than improve their capability (Baumeister, 1993; Rosenberg and Owens, 2001). Thus, people with low self-esteem have more difficulty succeeding in their careers and are more likely to experience problems such as Internet addiction (Bahrainian et al., 2014; Malik and Khan, 2015), drug and tobacco addiction (Pedersen et al., 2013), eating disorders (Rhea and Thatcher, 2013; Brechan and Kvalem, 2015), mental disorders, and even suicide (Brausch and Decker, 2014; Lakey et al., 2014).

Self-esteem is an internal personal characteristic that is difficult to change. Therefore, other methods of mitigating depression are needed. The results of the mediation effect analysis in the current study indicated that feelings of entrapment and defeat completely mediate the relationship between self-esteem and depression. This suggests that addressing entrapment and defeat status may help to protect transgender people from depression. In the terms of clinical significance, the current findings highlight the need for mental health support for transgender women. Transgender women are vulnerable to mental health problems and many transgender women have to hide their true selves from society, family, and friends. As these women experience substantial discrimination, particularly in economically underdeveloped areas, so it is necessary for health workers to provide psychological counseling and support. Transgender women who receive a psychological diagnosis of low self-esteem should receive help not only to improve their self-esteem and accept themselves, but also to address the frustrations and difficulties they experience. To help transgender women address these challenges, it is necessary to understand why they feel defeat and entrapment and how to solve these problems. We can do further research on it.

The current study involves several limitations that should be considered. First, because this was a cross-sectional study, caution should be exercised when drawing causal conclusions. However, the current findings may provide support for Beck’s cognitive theory of depression (Beck, 1987). Further investigations are needed, such as cohort studies and longitudinal studies, to obtain more evidence for the conclusions presented here. Second, self-reports measures were used regarding participants’ transgender status and participants answered the questions with the help of researchers. These factors may have led to reporting bias. However, the high level of confidentiality was a positive feature of the study. Information confidentiality agreements were signed with respondents to ensure non-disclosure of information. The investigator underwent professional training to ensure that the information obtained was as valid and accurate as possible. Third, the sample was not countrywide but was limited to transgender women sex workers in Shenyang and Guangzhou, as representative cities in the north and south of China, respectively. However, we believe that the sample was highly representative of transgender women sex workers across China, and so the findings could be used to develop guidelines for all transgender women sex workers in China. In addition, Meyer proposed that public policy can affect individuals’ ability to cope with minority stress: “Further research on minority stress must address these public policy and public health recommendations as well as barriers to their implementation.” However, political attitudes and social empowerment regarding the lesbian, gay, bisexual and transgender community have not substantially changed in China in recent years. Thus, further investigation of the situations faced by transgender people in different political environments is necessary to find better solutions, and to address public policy and public health recommendations, as well as barriers to their implementation.

## Conclusion

The current study investigated depression among transgender women sex workers in Shenyang and Guangzhou, China, examining the mediating effect of entrapment and defeat on the relationship between self-esteem and depression. The mediation effect model hypothesized that self-esteem is directly related to depression and indirectly mediated by feelings of entrapment and defeat. Low self-esteem is considered a high-risk factor for depression. The results of our mediation effect model revealed that the interaction between defeat and entrapment was non-significant and thus this interaction was omitted. Further analysis indicated that entrapment and defeat completely mediate the relationship between self-esteem and depression. This indicates that self-esteem affects depression via feelings of entrapment and defeat.

## Data Availability Statement

The data that support the findings of this study are available on request from the corresponding author. The data are not publicly available due to privacy or ethical restrictions.

## Ethics Statement

The studies involving human participants were reviewed and approved by the Ethics Committee of the School of Public Health of Shanghai Jiao Tong University, China. The patients/participants provided their written informed consent to participate in this study.

## Author Contributions

All authors contributed to the whole process of the research. RC and HW analyzed the data and wrote the manuscript. RC, HW, RS, SZ, LT, QS, and YS participated in the experimental design and data collection. ZW, JL, YW, and YC gave guidance on the experimental design and manuscript writing.

## Conflict of Interest Statement

The authors declare that the research was conducted in the absence of any commercial or financial relationships that could be construed as a potential conflict of interest.
